# Adherence to treatment and parents’ perspective about effectiveness of melatonin in children with autism spectrum disorder and sleep disturbances

**DOI:** 10.1186/s13034-023-00669-w

**Published:** 2023-10-27

**Authors:** Hadar Sadeh, Gal Meiri, Dikla Zigdon, Michal Ilan, Michal Faroy, Analya Michaelovski, Yair Sadaka, Ilan Dinstein, Idan Menashe

**Affiliations:** 1 Preschool Psychiatric Unit, Soroka University Medical Center, Beer-Sheva, Israel; 2 Azrieli National Center for Autism and Neurodevelopmental Research, Beer-Sheva, Israel; 3 Zusman Child Development Center, Soroka University Medical Center, Beer-Sheva, Israel; 4 Child Development Clinic, Ministry of Health, Beer-Sheva, Israel; 5 Psychology and Brain and Cognition Departments, Ben-Gurion University of the Negev, Beer-Sheva, Israel; 6 Department of Epidemiology, Biostatistics, and Community Health Sciences, Faculty of Health Sciences, Ben-Gurion University of the Negev, Beer-Sheva, Israel

**Keywords:** Autism spectrum disorder, Sleep, Insomnia, Drug effectiveness

## Abstract

**Objective:**

Melatonin is considered an effective pharmacological treatment for the sleep disturbances that are reported in > 50% of children with autism spectrum disorder (ASD). However, real-life data about the long-term course and effectiveness of melatonin treatment in children with ASD is lacking.

**Methods:**

In this retrospective cohort study, we assessed the adherence to melatonin treatment and parents’ perspective of its effect on sleep quality and daytime behavior in children with ASD via a parental phone survey of children in the Azrieli National Center for Autism and Neurodevelopment Research (ANCAN) database. Cox regression analysis was used to assess the effect of key demographic and clinical characteristics on treatment adherence.

**Results:**

Melatonin was recommended for ~ 8% of children in the ANCAN database. These children were characterized by more severe symptoms of autism. The median adherence time for melatonin treatment exceeded 88 months, with the most common reason for discontinuation being a lack of effectiveness (14%). Mild side-effects were reported in 14% of children, and 86%, 54%, and 45% experienced improvements in sleep onset, sleep duration and night awakenings, respectively. Notably, melatonin also improved the daytime behaviors of > 28% of the children. Adherence to treatment was independently associated with improvements in night awakenings and educational functioning (aHR = 0.142, 95%CI = 0.036–0.565; and aHR = 0.195, 95%CI = 0.047–0.806, respectively).

**Conclusions:**

Based on parents’ report, melatonin is a safe and effective treatment that improves both sleep difficulties and daily behavior of children with ASD.

**Supplementary Information:**

The online version contains supplementary material available at 10.1186/s13034-023-00669-w.

## Introduction

Sleep disturbances are highly prevalent in children with autism spectrum disorder (ASD), with an estimated prevalence ranging between 30 and 90% [1–4]. These sleep disturbances, which may occur from early life into adolescence and adulthood [5–7], include, but are not limited to, delayed sleep onset, multiple night awakenings, early morning awakenings, low sleep efficiency, delayed circadian phases, and shortened total sleep time [4–6]. Children with ASD who experience sleep disturbances usually manifest more severe core symptoms of ASD, such as poorer social capabilities, more restricted-repetitive behaviors, and more communication and language problems than their non-ASD counterparts [7–9]. Moreover, sleep problems in children with ASD are also associated with the manifestation of other behavioral problems, such as irritability, aggression, anxiety, affective problems, hyperactivity, attentional deficits and poorer functional and adaptive skills [6, 7, 13, 14].

Melatonin is an endogenous hormone produced in the pineal gland [10]. Its secretion is regulated through light-dark cues, by the biologic clock, the supra-chiasmatic nucleus (SCN) in the hypothalamus. It plays a role in various physiologic functions, including sleep promotion and circadian modulation through feedback activity on the SCN and peripheral tissues [10]. Due to its key function in the circadian rhythm, melatonin is used to treat various forms of sleep problems [11], including those manifested by children with ASD [12, 13].

Melatonin is usually prescribed for treating sleep latency in children with ASD, but it has also been reported to increase total sleep duration and sleep efficiency in those children [19, 20] and to reduce night awakenings (particularly for the prolonged-release formulation) [14, 15]. Moreover, melatonin treatment before bedtime has been found to improve daytime behavior and core symptoms of children with ASD [16–18]. Importantly, melatonin is considered to be safe, with only a few transient or mild reported side effects, such as early morning awakenings, morning drowsiness, headache, mood swings and irritability [14, 18–20].

Despite the evidence regarding the positive effect of melatonin treatment on sleep disturbances in children with ASD, only 7–13% [12, 18] of children with ASD are prescribed melatonin for sleep problems. In addition, real-life and long-term data on the course and effectiveness of melatonin treatment are sparse. Finally, the clinical characteristics associated with adherence to melatonin treatment are vague. For these reasons, we studied the real-life prevalence, course, and effectiveness of melatonin treatment in a representative sample of children with ASD in Israel.

## Methods

### Study design

We conducted a retrospective cohort study of children diagnosed with ASD who are registered in the database of the Azrieli National Center for Autism and Neurodevelopment Research (ANCAN), Israel [21. Inclusion and exclusion criteria for the study sample are shown in Figure S1, available online. We included children whose database records contained an indicator or recommendation for melatonin use and whose parents signed an informed consent form allowing us to contact them for research purposes. We contacted the parents of those children by phone and asked them to answer a brief survey about their experience with the melatonin treatment. We excluded children without contact details (e.g., phone number or email address), those whose parents refused to participate in the study, and those whose parents indicated that the children had not been given melatonin or had taken it for less than a week.

### Data collection

We assessed treatment adherence and parents’ perspective about the effect of melatonin treatment using the above-described parental phone survey. The survey (which is presented in Supplementary file 1, available online) comprised two parts: (1) eight questions, adopted from other surveys [22, 23], that include details about melatonin dosages, usage patterns (time and frequency), duration of treatment, reason for stopping or continuing treatment, side effects, and source of the medicine; and (2) eight questions that were designed to assess the child’s sleep and general daily functioning and behavior, on a scale of 1 to 5 (1—major negative effect; 2—minor negative effect; 3—no effect; 4—minor positive effect; and 5—major positive effect), as described previously [22–24]. Adherence to treatment was assessed using data about treatment initiation and treatment cessation/continuation based on questions 1 and 3 in the survey (Supplementary file 1, available online ).

Sociodemographic, behavioral, and clinical data about participating children were obtained from the ANCAN database. Clinical variables included the Autism Diagnostic Observation Schedule™, Second Edition (ADOS®-2) [25] comparison score, the DSM-5 ASD severity levels, the preschool language scale, fourth edition (PLS4) total score [26], a cognitive score based on either the Bayley Scales of Infant and Toddler Development-third edition (Bayley-III) [27] or the Wechsler Preschool and Primary Scale of Intelligence—version three (WPPSI-III) [28], The General Adaptive Composite (GAC) score of the Adaptive Behavior Assessment System-II (ABAS-II) [29], and data about the child’s sleep quality based on the Children’s Sleep Habits Questionnaire (CSHQ) [30]. Additional data about chronic comorbidities and medication use were obtained from the children’s medical files.

### Statistical analysis

Standard univariate statistics were used to assess differences in sociodemographic and clinical characteristics between the study cohort (i.e., children who were reported to use short-release melatonin or were recommended to use it) and other children in the ANCAN database as well as between children whose parents reported melatonin had/did not have an effect on their sleep. Kohen’s kappa was used to assess the concordance between different effects of melatonin treatment. Kaplan-Meir and Cox regression analyses were used to assess sociodemographic and clinical variables associated with adherence to melatonin treatment. Statistical significance in all these analyses was determined at a p-value of < 0.05. All analyses were carried out using SPSS Software Version 28.

## Results

Among the 1,355 children with ASD in the ANCAN database (November 2021), there an indication for melatonin recommendation or use for only 107 (8%) children (Figure S1, available online). A comparison of the sociodemographic and clinical characteristics between children with ASD who were treated with melatonin and those who were not is presented in Table 1. Children who were given melatonin were diagnosed, on average, six months earlier than children who were not (35.1±15.3 months vs. 41.4±19.3 respectively; p-value = 0.002). Children who were treated with melatonin had more severe ASD symptoms, according to both DSM-5 criteria and the ADOS-2 comparison score, and they also had lower cognitive and adaptive behavior abilities (Table 1). As expected, children who were treated with melatonin had more severe sleep disturbances, according to the CSHQ total and subscales scores, compared to their counterparts who were not.

**Table 1:** Comparison of Sociodemographic and Clinical Characteristics between Children with ASD with and without a Melatonin Recommendation

Variable		Melatonin recommended (n = 107)	Melatonin not recommended (n = 1248)	P-value
Age at diagnosis in months (mean±SD)		35.1 (+-15.3)	41.4 (+-19.3)	**0.002** ^**a**^
Sex (% male)		88 (82%)	989 (79%)	0.561^ C^
Ethnicity (% Jewish)		89 (84%)	1001 (82%)	0.705^ C^
ADOS-2 comparison score (mean±SD)		7.7 (+-2.2)	6.9 (+-2.2)	**0.002** ^**a**^
DSM-5 severity level (A criteria)	Requiring support	10 (10%)	234 (21%)	**0.004** ^**c**^
	Requiring substantial support	42 (41%)	496 (44%)	
	Requiring very substantial support	50 (49%)	391 (35%)	
DSM-5 severity level (B criteria)	Requiring support	14 (13%)	290 (23%)	**< 0.001** ^**c**^
	Requiring substantial support	42 (39%)	575 (46%)	
	Requiring very substantial support	45 (42%)	263 (21%)	
Cognitive score (mean±SD)		71.6 (+-18.6)	76.1 (+-19.9)	0.067^a^
ABAS GAC score (mean±SD)		57.3 (+-9.5)	67.3 (+-15.9)	**< 0.001** ^**a**^
PLS score (mean±SD)		63.8 (+-24)	62.5 (+-32)	0.780^a^
CSHQ	Total sleep score (mean±SD)	51.8 (+-10.3)	46.1 (+-9.3)	**< 0.001** ^**a**^
	Sleep onset delay (mean±SD)	2.3 (+-0.8)	1.8 (+-0.8)	**< 0.001** ^**a**^
	Night wakings (Mean±SD)	5.2 (+-1.8)	4.7 (+-1.7)	**0.026** ^**a**^
	Sleep duration (mean±SD)	5.1 (+-1.8)	4 (+-1.5)	**< 0.001** ^**a**^

Note: ^a^ T- test; ^b^ Fisher’s exact test; ^c^ Chi-square test (1 df)

### Characteristics of the melatonin-treatment cohort

**Fig. 1:**
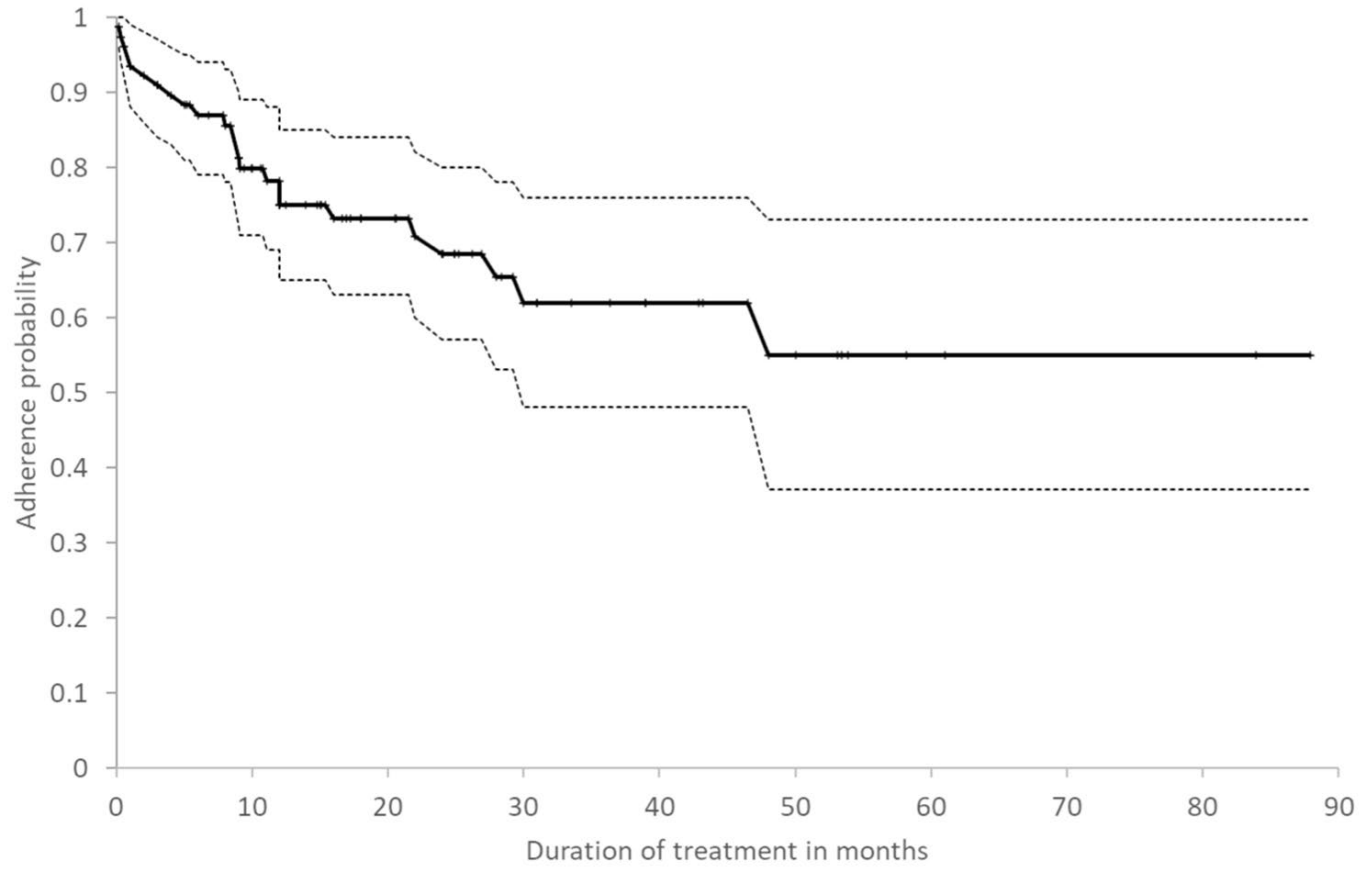
Kaplan-Meier Curve of Melatonin Treatment Adherence over Time. The cumulative adherence probability to melatonin treatment over time is depicted by a continuous black line with 95% confidence intervals in black dashed lines

Overall, the parents of 78 children (73%) for whom there was an indication for melatonin recommendation or use completed our phone survey and reported adherence to melatonin treatment for at least one week. Notably, the median adherence time for those children exceeded 88 months (Fig. 1), with 26 children (33.5%) discontinuing the treatment for various reasons, including lack of effectiveness (14.1%), natural improvement (8.9%), loss of effectiveness (7.7%), side effects (3.8%), starting other medications (3.8%), child’s refusal (2.6%), doctor’s recommendation (2.6%), medication cost (1.3%), or starting a behavioral sleep intervention (1.3%) (Table S1, available online).

The sociodemographic and clinical characteristics of the children who were treated with melatonin, and the association of these characteristics with treatment discontinuation, are presented in Table 2. None of these characteristics was significantly associated with treatment discontinuation, although the use of other psychoactive drugs concurrently with melatonin treatment was marginally associated with a lower probability of treatment discontinuation (HR = 0.452, 95%CI = 0.194–1.052, respectively). Characteristics associated with melatonin treatment and their effect on treatment discontinuation are also presented in Table 2. The age at treatment initiation ranged between 1.6 and 9.6 years, and the mean final melatonin dosage was 3.9 (±2.7) mg. Most of the parents (77%) gave the treatment every day before bedtime, while the others gave melatonin only on school days, or when the child had serious sleep onset difficulties. Interestingly, the parents of 14 children (18%) purchased an imported over-the-counter (OTC) formulation of melatonin, without prescription. The parents of 11 children (14%) reported mild side effects of melatonin treatment, including crying, irritability/hyperactivity, morning drowsiness, rash, vomiting, fever, increased appetite, and abdominal pain (Table S1, available online). None of the treatment characteristics or its side effects was significantly associated with treatment discontinuation.

**Table 2:** Characteristics Associated with Discontinuation of Melatonin Treatment

Variables			All	HR for treatment discontinuation	95% CI	P-value
Sociodemographic and clinical characteristics						
Sex, n (%) males			67 (86%)	1.165	0.397–3.413	0.781
Ethnicity, n (%) Jewish			62 (79%)	0.864	0.294–2.544	0.791
Age at diagnosis in months (mean ± SD)			34.3+-15.6	0.875	0.618–1.239	0.452
ADOS-2 comparison score (mean ± SD)			7.4+-2.2	1.071	0.875–1.312	0.505
DSM-5 severity level (A criteria), n (%)		Requiring support	0 (0%)	0.539		
		Requiring substantial support	36 (46%)		0.238–1.218	0.137
		Requiring very substantial support	39 (50%)			
DSM-5 severity level (B criteria), n (%)		Requiring support	4 (5%)			
		Requiring substantial support	35 (45%)	1.060	0.457–2.458	0.892
		Requiring very substantial support	36 (46%)			
PLS score (mean ± SD)			63.2+-24.3	0.995	0.975–1.015	0.642
Cognitive score (mean ± SD)			71.9+-18	1.020	0.993–1.047	0.150
ABAS GAC score (mean ± SD)			57.3 +-10.3	0.992	0.929–1.060	0.820
Any medical comorbidity, n (%)			38 (49%)	0.669	0.293–1.530	0.341
Any neuro-developmental comorbidity,^a^ n (%)			9 (11%)	1.162	0.345–3.909	0.809
Any emotional-behavioral comorbidity,^b^ n (%)			7 (9%)	0.038	0.000-6.811	0.217
ADHD, n (%)			25 (32%)	0.605	0.245–1.494	0.276
Any other psychoactive medications,^c^ n (%)			37 (47%)	0.452	0.194–1.052	0.066
Antipsychotic medications, n (%)			19 (24%)	0.729	0.272–1.955	0.530
Stimulant medications, n (%)			15 (19%)	0.796	0.291–2.178	0.657
Treatment characteristics						
Age at treatment beginning in months (mean±SD)			51.9 (+-20)	1.118	0.890–1.404	0.336
Final dosage in mg (mean±SD)			3.98 (+-2.7)	0.920	0.769–1.102	0.367
Treatment course	Times per week (mean±SD)		6.3 (+-1.5)	1.385	0.843–2.276	0.199
	Continuity (no cessation of more than several days), n (%)		63 (81%)	1.798	0.535–6.040	0.342
Source of medication—imported, as dietary supplement, n (%)			14 (18%)	0.490	0.145–1.656	0.251
Any side effects, n (%)			11 (14%)	1.919	0.710–5.188	0.199

Note: ^a^ including- epilepsy, Down’s syndrome, global and motor developmental delay, hydrocephalus; ^b^ including- anxiety, enuresis, encompresis, oppositional-defiant disorder; ^c^ including- Prothiazine, anti-psychotics, anti-convulsants, SSRI, stimulants, cannabis

### Effect of melatonin treatment

**Fig. 2:**
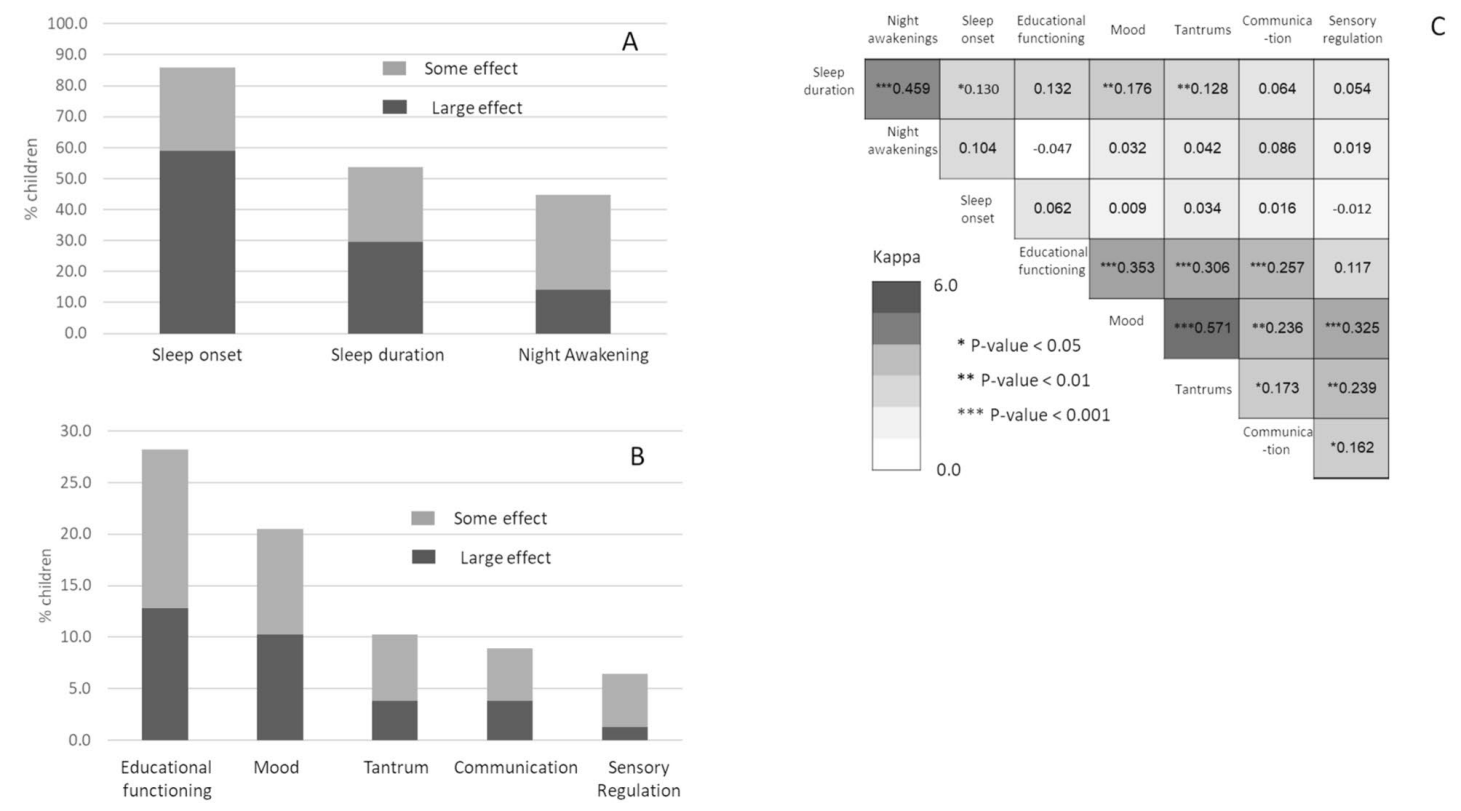
Effect of Melatonin on Sleep and Daytime Behaviors of Children with ASD. **(A) &** (**B**) Rates of children whose parents reported an effect of melatonin treatment on their sleep parameters (**A**) and daytime behaviors (**B**). (**C**) Kohen’s kappa coefficients for the concordance between different effects of melatonin treatment

The parents of 70 children (90%) reported that melatonin improved their children’s sleep, with 86%, 53%, and 45% reporting that melatonin treatment influenced the sleep onset, sleep duration, and night awakenings, respectively (Fig. 2A). In addition, the parents of 27 children (35%) reported that melatonin had an additional effect on their children’s daytime behavior, with better educational functioning, improved moods, reduction in tantrums, better communication abilities and better sensory regulation being reported in 28%, 21%, 10%, 9%, and 6% of children, respectively (Fig. 2B). Of note, a moderate and statistically significant concordance was seen between the effect of melatonin treatment on different sleep and daytime behaviors, with the most significant associations being between sleep duration and night awakening (kappa = 0.459; p-value < 0.001), and between tantrum reduction and mood improvement (kappa = 0.571; p-value < 0.001) (Fig. 2C).

Finally, both univariable and multivariable Cox regression models were used to assess the association between the reported effect of melatonin treatment on sleep and daytime behavior and treatment adherence (Table 3). Improvements in nighttime awakening, sleep duration and educational functioning were significantly associated with a decreased likelihood to discontinue melatonin treatment (HR = 0.208, 95%CI = 0.071–0.610; HR = 0.376, 95%CI = 0.161–0.878; and HR = 0.271, 95%CI = 0.080–0.91, respectively) in the univariate analysis. However, because of the relationship between the effects of melatonin treatment on sleep duration and night awakenings (see Fig. 2C), only the effects of night awakenings and educational functioning remained significantly associated with treatment adherence in the multivariate analysis (aHR = 0.142, 95%CI = 0.036–0.565; and aHR = 0.195, 95%CI = 0.047–0.806, respectively).

**Table 3:** Characteristics of Treatment Effectiveness and its Effect on Adherence

Reported effect ^a^		N (%)	Univariate HR for treatment cessation	95% CI	p value	Multivariate HR for treatment cessation	95% CI	p-value
Effect on sleep quality								
Sleep onset		67 (86%)	0.535	0.199–1.435	0.214	1.055	0.333–3.343	0.928
Sleep duration		42 (54%)	**0.376**	**0.161–0.878**	**0.024**	1.230	0.411–3.686	0.711
Night awakening		35 (45%)	**0.208**	**0.071–0.610**	**0.004**	**0.142**	**0.036–0.565**	0.006
Effect on daily behavior								
Educational functioning		22 (28%)	**0.271**	**0.080–0.913**	**0.035**	**0.195**	**0.047–0.806**	0.024
Mood		16 (21%)	0.468	0.139–1.573	0.220	0.191	0.005–7.344	0.374
Temper tantrums		8 (10%)	0.290	0.039–2.152	0.226	2.212	0.058–83.932	0.669
Communication		7 (9%)	0.412	0.056–3.050	0.385	2.764	0.249–30.628	0.407
Sensory regulation		5 (6%)	1.093	0.256–4.655	0.905	4.119	0.119-142.282	0.434

Note: ^a^ at least some effect

## Discussion

To the best of our knowledge, this is the first study to examine the real-life adherence to melatonin treatment and its effect on both sleep and daytime behaviors of children with ASD. In our sample, only 8% of ASD children were advised to take melatonin to ameliorate their sleep problems. This 8% prevalence of melatonin treatment in children with ASD is slightly higher than the 3.8% melatonin usage reported in a previous study in the same population [31], but is similar to the reported usage of melatonin in two meta-analyses [29, 38]. Such a relatively low prevalence of melatonin usage stands in contrast to the > 50% prevalence of sleep disturbances reported in children with ASD [1, 2, 4, 37] and is especially surprising in light of the significant effects of melatonin on the sleep quality and daytime behavior in these children, as is evident in our study and others [15, 18]. One possible explanation for this enigma lies in the concerns of some pediatricians about the side effects of and tolerance to melatonin, which may discourage them to recommend its use [38, 39]. Another possible reason may stem from the known under-diagnosis by clinicians of sleep problems in children with ASD [32–34]. Finally, it should be noted that it is not impossible that the use of imported OTC melatonin in our sample might be higher than that reported by the parents.

The children who were treated with melatonin were characterized by more severe symptoms of autism than the other children in our cohort. This may be attributed to the known association between sleep disorders and the severity of autistic symptoms, which may motivate parents to seek treatment for their children [7, 10, 11]. Adherence to melatonin treatment was also slightly associated with the severity of autistic symptoms. hadt. This could be due to the fact that children with more severe autistic symptoms suffer from a more severe melatonin deficiency [35–37] and consequently derive greater benefit from the melatonin treatment.

The treatment with melatonin reported in this study was generally administered according to the accepted guidelines, which recommend administration once a day before bedtime [13]. Furthermore, most parents also complied with the recommended dose of up to 6 mg—any further increase in the dose has been reported to be ineffective in these children [13, 38]. Indeed, the lack of an association between final melatonin dose and treatment adherence in our cohort further supports the notion that higher doses of melatonin do not guarantee a better effect.

Approximately one fifth of the children who were given melatonin in our sample took an OTC formulation that was purchased online. Such a significant use of OTC melatonin may be attributed to the relatively easy accessibility to imported OTC melatonin and the fact that melatonin treatment was not covered by the HMOs at the time of the study. Interestingly, in terms of reported adherence and effectiveness, the OTC formulation of melatonin taken by children in the study was comparable to the prescribed melatonin, despite the remarkable variation in melatonin content in such OTC formulations [39] and the recommendation for using prescription-only melatonin preparations [13]. Unfortunately, we did not have information about the different types of OTC melatonin taken by the children in this study, so we could not explore differences between specific types of OTC melatonin.

Melatonin has been shown to be a relatively safe treatment, and only 14% of parents in this study reported a few mild side effects that did not affect adherence to treatment. Such minor side effects have also been reported in the literature [14, 18–20], although the rates of side effects were a little lower in our study, possibly because of a recall bias. Of note, despite the relatively long follow-up for most of the children in our study, some of the reported long-term side effects of melatonin treatment, such as precocious puberty [40] could not be observed in our sample, due to relatively young age at which treatment was initiated in most of the children.

We showed that melatonin treatment improved sleep onset in most (86%) of children, but the factors that were mostly associated with the treatment adherence were improvement in night awakenings and sleep duration. Furthermore, some parents reported that melatonin treatment also improved the daytime behaviors of their children, with an improvement in educational functioning being independently associated with treatment adherence. These findings reflect the broader effect of melatonin treatment beyond its direct effect on sleep onset. Importantly, the melatonin used in our study had a short-term effect and was therefore less effective in affecting sleep problems other than sleep onset. It is possible that other formulations of melatonin (e.g., prolonged-release formulation [41]) or other sleep medications may improve adherence to melatonin administration in these children.

The results reported in this study should be interpreted in the context of the following limitations. First, despite the large cohort of children at the ANCAN database, only 8% were treated with melatonin, a fact that significantly reduced the study sample size and the statistical power of analyses exploring the melatonin effectiveness. Second, the data on the reported effects of melatonin treatment were obtained through parental surveys, which may be affected by both recall and information biases. Third, we didn’t have data on the socioeconomic status of participants, which may affect both melatonin use and parents’ perspective. Nevertheless, we did have data on the ethnic background of participants, which is strongly associated with the socioeconomic status in the studied population [42, 43]. Forth, we did not have data regarding the exact formulation of melatonin used by each child and therefore we could not compare the effect of different melatonin formulations on treatment adherence, sleep quality and daytime behavior. Finally, the number of participating children was relatively small and limit our ability to identify factors with small effect on adherence to melatonin treatment.

## Conclusions

Our results indicate a significant adherence to melatonin treatment and positive parents’ perspective on its effect on both sleep quality and daytime functioning in children with ASD. Future prospective studies and clinical trials are needed to objectively quantify these reported effects of melatonin treatment in children with ASD.

### Electronic supplementary material

Below is the link to the electronic supplementary material.

Supplementary Material 1

## Data Availability

available upon reasonable request.

## List of abbreviations

ASD: autism spectrum disorder
SCN: supra-chiasmatic nucleus
ANCAN: Azrieli National Center for Autism and Neurodevelopment Research
OTC: over-the-counter
